# Stress in Balancing Work and Family among Working Parents in Hong Kong

**DOI:** 10.3390/ijerph19095589

**Published:** 2022-05-04

**Authors:** Qiqi Chen, Mengtong Chen, Camilla Kin Ming Lo, Ko Ling Chan, Patrick Ip

**Affiliations:** 1 Department of Social Work, School of Sociology and Anthropology, Xiamen University, Xiamen 361005, China; qqchen@xmu.edu.cn; 2 Department of Applied Social Sciences, The Hong Kong Polytechnic University, Hung Hom, Hong Kong; jenna.mt.chen@polyu.edu.hk (M.C.); camilla.lo@polyu.edu.hk (C.K.M.L.); 3 Department of Paediatrics and Adolescent Medicine, LKS Faculty of Medicine, The University of Hong Kong, Pokfulam, Hong Kong; patricip@hku.hk

**Keywords:** work and family balance, stress, satisfaction, childcare, elderly care

## Abstract

Work-life imbalance might lead to detrimental outcomes, including family dissatisfaction, poor performance in the workplace, and poor mental and physical health. This population-based study aims to explore the situation and trends in regard to work-life balance among working men and women in 2017, with a special focus on the stress experienced in work and personal lives. Descriptive analysis and multiphase regression are used to explore the associations of work-life imbalance with individual and family factors. Males’ satisfaction with the amount of time spent at work was most significantly related to the level of work-life stress. Both males’ and females’ satisfaction with work life, family life, and the amount of time spent at work and with family were all negatively related to the level of work-life stress. Participants who were not in marital or cohabiting status reported significantly higher levels of work-life stress. Participants who had childcare support reported higher levels of work-life stress than those who looked after their children by themselves or their partners. A similar pattern was found among participants involved in elderly care. This study provides insight into family policy that could promote balance in professional and personal life and relationships.

## 1. Introduction

Time, as a resource for working, resting, and caring for dependents, has been proposed as a social determinant of health [1]. Lack of time for rest is reported to be associated with unhealthy behaviors, such as alcohol consumption, smoking, and lack of exercise [2,3]. Not having time to rest from work could lead to poor physical and mental health status, such as stress, sleeping problems, and elevated blood pressure [4]. According to a recent report by the World Health Organization (WHO) and the International Labour Organization (ILO), at least 745,000 deaths from stroke and ischemic heart disease in 2016 were related to long working hours, an increase of 29% on the corresponding figure for 2000 [5]. The lack of time for personal life due to excessive working hours may lead to work–life imbalance [3].

Recent studies have disclosed that balance describes the emotional aspects of work and family life that are important for individuals [6]. The balance between work and life has been identified as providing ample benefits, including enhanced job satisfaction and commitment [7], reduced absence due to sickness or mental illness, reduced turnover intention, and improved job performance [8]. Researchers have also suggested that work–life imbalance may play a moderating role in the relationship between working hours and health-related well-being [9]. Emerging economies in Asia have grown significantly in the past decades; this growth has contributed substantially to the global economy but has also shifted individuals’ social and economic focus more towards work [10]. The WHO has suggested that long working hours are a prevalent occupational risk factor for a large number of deaths from ischemic heart disease and stroke [5].

Work–life balance currently entails a variety of measurement approaches. We referred to some of the theoretical definitions [11,12] as the framework and measured individuals’ subjective satisfaction and stress of balance, objective measures of time spent in each domain, and the perception on the time distribution on work and life. Work–life balance is defined here as the struggle to meet role demands, which is often determined by factors related to employment duties and family responsibilities [12]. Conservation of Resources (COR) theory [13] emphasizes an individual’s drive to protect their resources. Work–life balance could be deemed as a possessed time resource to allocate, where people need to choose where to allocate the time with repeated work–life demands in their daily lives [14]. The decision in establishing the balance between work and life is often informed by personal experiences and social circumstances, such as financial incentives, personal interests, gender dynamics, and cultural context [15]. For example, gender is often imbedded within work–family interactions, where men are more likely to engage in breadwinning while women are more likely to bear the burden of childrearing and household labor [16]. Although the number of dual-earner households has been increasing across the globe in the past decades, women are still expected to fulfil domestic duties, regardless of their employment [15], and men are still found to not actively participate in housework [17]. Studies have indicated that children tend to seek attention by interrupting their mothers, which makes mothers’ time more fragmented [18]. This preference could result in women being more overwhelmed and experiencing greater distress in their work–family role [19].

Work–family imbalance resulting from the ongoing strain of family poverty, long working hours, and heavy work overload could lead to stress and chronic illness for working parents [20]. Some studies have described the relationship between the higher risk of work–family imbalance and worse health status due to job insecurity caused by temporary employment or to high job demands [21]. This could be explained by the COR theory that individuals appraise the resources required to meet those demands, which could ultimately result in decisions about how to allocate their personal resources [22]. This subjective appraisal could also lead to expectancy–outcome violation and dissatisfaction or conflict among life pursuits [14,22]. Therefore, resource allocation could be highly individualistic and related to individual characteristics and beliefs.

COR theory also suggests that individuals are motivated to expend resources they prefer and minimize that on the required activities that are required [14]. For example, educational attainment is positively related to employment and wages, which leads to an indication of a gender convergence in many developed countries [12]. The assimilation of women in the workforce can increase family income and the independence of women [23]. The increasing workload experienced by both genders could significantly affect family life; however, women pressurized in work reported more difficulties in taking care of family, specifically mothers with younger children, while men often feel more satisfied at work at the cost of ignoring family [24]. Studies also showed that women who have younger children outperformed women with older children [24]. Existing literature has argued that compared with less educated males, more highly educated males are more likely to do housework [12]. Women within couples that are more highly educated spend less time on housework, which may intensify the gender conflict in regard to the division of work and life within households [19]. Conflicts within a family may ruin working parents’ marital satisfaction and intensify the parental burden [6].

The speed and intensity of the competitive work environment in Hong Kong is famed for its efficiency and lively spirit but has also produced workers with significant stress, owing to long working hours and demands for greater productivity [25,26,27]. Filial piety within the hierarchical family structure in Hong Kong also includes parents’ obligation to care for their children and the elderly members of their family, and this is a contributory factor to workers placing a strong emphasis on their careers and spending long hours at work for the success of their family [28,29]. On the other hand, the perception of job insecurity is a major factor behind long working hours in Hong Kong, where commitment to the work role is a means to providing financial security for one’s family [30]. Thus, sacrificing family time for work is viewed as being a benefit for the family in terms of gaining long-term benefits, such as being able to pay off a mortgage and meet other major household costs [15]. Researchers have also suggested that individuals may internalize cultural influence as a structure of habits and as a personal variable in the cognitive appraisal process [22]. For example, a comparative study of work–family stressors contrasting Anglicized nations and China, found that anglicized individuals demonstrate a stronger positive relation between work hours and work–family stressors, while the Chinese tend to relate being married and having more children to high levels of well-being.

As reviewed above, individual differences and cultural beliefs could influence the appraisals of resources and the response to work–life demands. Thus, identifying with a particular role may play an important part in determining the level of resources to expend in that role. Despite the growing body of evidence from work–life balance research in Western countries [31], research in this area in Hong Kong focused more on a macro level, such as the impacts of societal and cultural norms [25,32,33], and the family-friendly policy [34] on work–life balance [15]. There has been limited study reporting individual or family characteristics on this issue [35]. This study aims to provide updated insights into how individual and family factors influence perceptions of the work–life interface. We hypothesize that (a) family-life stress differs by gender; (b) the family-life stress individuals experience varies by marital status, and (c) individuals’ satisfaction status with work and life are related to stress from work–life imbalance.

## 2. Materials and Methods

### 2.1. Study Design and Procedure

Data from the Family Survey carried out by the Family Council in 2017 were used to investigate various predictors of the current situation among economically active families in Hong Kong. The surveys were commissioned by the Home Affairs Bureau of the Government of the Hong Kong Special Administrative Region on a biannual basis beginning in 2011. The survey provided updated and empirical information regarding the changes in family functioning, social support networks, the perception of family development, and the awareness of family-related programs. The survey was designed to provide insight into the changes in Hong Kong families, including the challenges they face and the types of support they require. The study process has been approved by the ethical committee of the authors’ affiliated institution. The survey purposively sampled all persons aged 15 or above residing in Hong Kong, regardless of gender, sexual orientation, religion, geography, ability, language, and culture, as the target population. Initially, 6500 living quarters were randomly sampled from the Hong Kong Census and Statistics Department Frame of Quarters; 3000 of the quarters were successfully enumerated based on the criteria of having eligible respondents aged 15 or above [36]. The estimate level is within the range of ±2.2 percentage points at 95% confidence level. The response rate of the surveys was 63.5%. An effective sample size of 1300 respondents (M = 44.37, SD = 13.05) was obtained from the Family Survey. These 1300 respondents were economically active during the survey period and provided sufficient data in the questionnaire for analysis on this topic.

### 2.2. Measures

#### 2.2.1. Stress from Balancing Work and Family Life

Information on views regarding balancing work and family was assessed with a single item: “Your level of stress in regard to balancing your work and family life.” The questions included a question on participants’ perceived level of stress in balancing work and family (0 = “Refuse to answer,” 1 = “No stress at all,” 2 = “Not very much stress,” 3 = “Some stress,” 4 = “A great deal of stress”). A higher score indicated higher levels of stress experienced from meeting the demands of balancing work and family life.

#### 2.2.2. Satisfaction with Work, Family, and Time Spent at Work and with Family

Overall satisfaction with time spent at work and with family among the respondents was measured with three items: satisfaction with family life (“Are you satisfied with your family life?”), satisfaction with work life (“Are you satisfied with your work life?”), and satisfaction with the amount of time spent at work and with family (“Thinking about the time you spend at work and with your family members, are you satisfied?”). Items were rated separately on Likert scales ranging from 1 (very dissatisfied) to 5 (very satisfied). A higher score indicated greater satisfaction on specific items among the participants.

#### 2.2.3. Availability of Assistance from Family Members

The participants were asked to assess whether their family members were supportive if problems were encountered. The availability of assistance from family members was considered in six circumstances: “when you are sick,” “when you need to make an important decision,” “when you are depressed and upset,” “when you are unemployed and cannot get a job,” “when you have financial problems,” and “when you want to share your happiness with your family members.” Level of support or helpfulness was rated on a six-point Likert scale (ranging from 1 = not helpful or supportive to 6 = helpful or supportive). A higher score indicated a greater level of assistance one could get from family members. The reliability of this six-item scale is good (Cronbach’s α = 0.92).

### 2.3. Demographic Characteristics

Participants were asked to provide demographic information—gender, age, individual monthly income, education level, marital status, whether or not they had children under 18, whether or not they had elderly relatives aged over 65, average number of working hours per week, and the major caregiver of their children and elderly relatives—using self-constructed items. Educational attainment was coded into three categories: primary education or lower, secondary education, and postsecondary education or above. Marital status was grouped into single (including divorced or separated, never married, and widowed) and married (including cohabiting with a partner). Major caregiver of children included four categories: childcare support (including grandparent, relative, maids, and others), care provided by self or partner, children do not need care provision, and no children. Major caregiving for elderly relatives was categorized into elderly care support (relative, maids, and others), care provided by self or partner, elderly relatives do not need care provision, and no elderly relatives over 65.

### 2.4. Statistical Analysis

This study employed data on perceived work–life balance, stress, and satisfaction among dual-working parents in Hong Kong in 2017. Descriptive statistics were first used to summarize the demographic characteristics of the survey participants, including age, individual monthly income, education attainment, marital status, total working hours per week, whether or not they had children or elderly relatives, and the major caregiver of their children and elderly relatives. Descriptive analyses were also used to calculate the availability of assistance, the level of stress from efforts to meet the demands of work and family life, and work and family life satisfaction. All the demographic and predictor variables were compared by gender using chi-square tests or t-tests where appropriate. We conducted a series of hierarchical linear regression analyses to estimate the associations among demographic and family characteristics, availability of assistance, work and life satisfaction, and stress from seeking balance in work and family life. This approach could enable the examination of the relative contributions of each domain of predictors. Specifically, in the first regression model, demographic characteristics and working hours were entered as independent variables to control for these determinants of stress from seeking a work–life balance. The second model included the major caregivers of children and elderly relatives and the availability of assistance measures, respectively, as higher-level predictors in an additive and gradual manner, with other covariates adjusted for. The final regression model included variables in all three satisfaction domains. Stress from meeting the demands of balancing work and family life was the dependent variable in all phases. The same set of analyses was conducted separately by gender to evaluate differences in the impacts of the determinants on work–life stress. The statistical level of 0.05 by two-tailed tests was set as significant, and all of the above tests were performed using SPSS version 25.0 (IBM Corp., Armonk, NY, USA).

## 3. Results

### 3.1. Demographic Characteristics and Explanatory Variables by Gender

Table 1 presents the respondents’ demographic characteristics and the explanatory variables. Gender differences were observed in some parameters. The total sample comprised 1300 eligible participants who completed the survey, 52.5% (n = 683) of whom were males and 47.5% (n = 617) females. The mean ages of the fathers and mothers were 44.76 (SD = 13.56) and 43.93 (SD = 12.47) years, respectively. In terms of monthly income, there were more males than females (*p* < 0.001) in all salary bands, except for the less than HKD 10,000 (approximately USD 1282) category, in which there were more females than men. In terms of education, 26.6% of the overall sample had completed postsecondary education and 12.9% had received a primary education or lower. No gender difference was found in regard to educational attainment.

Relatively more of the participants (55.1%) were married or cohabited with a partner, with more male than female participants reporting a married status (*p* < 0.05). Regarding child and elderly dependents, 54.9% of the participants reported having a child under 18 and 61.4% reported having an elderly relative aged over 65 to take care of. A gender difference was found in regard to having a child dependent, with more females than males reporting that they had a child to take care of (*p* < 0.01). The respondents worked 44.90 h (SD = 14.74) per week on average, and the male respondents (47.59 h) reported significantly longer working hours than the female respondents (41.91 h). Among those who had children and elderly relatives at home, most (24.3%) reported that they or their partners took care of their children (24.3%) and elderly relatives (28.5%); only 15% had childcare support and 19.5% had elderly care support from maids or relatives.

Table 1 also presents the work–life-balance-related variables, including availability of assistance (M = 4.40, SD = 0.96), level of stress resulting from efforts to meet the competing demands of work and family life (M = 2.70, SD = 0.74), satisfaction with the amount of time spent at work and with family (M = 3.60, SD = 0.73), and satisfaction with work life (M = 3.66, SD = 0.63) and family life (M = 3.89, SD = 0.68). A *t*-test indicated no significant gender difference in all the above items.

### 3.2. Stress in Balancing Work and Family by Gender

To examine the associations between demographic characteristics, level of work–life stress, and satisfaction with work and family life, we conducted a series of hierarchical regression analyses (see Table 2). In our analysis, we examined one variable at a time while controlling for all other variables. Model 1 showed that the participants who were not in a marital or cohabiting relationship (i.e., divorced/separated, never married, or widowed) reported significantly higher levels of work–life stress from meeting the demands of work and family life (B = −0.166, *p* < 0.001); this applied in the case of both the male and female respondents (Bs ranged from −0.162 to −0.178, all *p*s < 0.05). Both the male and female participants with longer working hours reported higher levels of work–life stress (Bs ranged from 0.010 to 0.011, all *p*s < 0.001).

In Model 2, when we added the major caregivers of children and the elderly and availability of assistance measures into the analysis, we found that compared with those with no caring duties, the participants who had children at home to take care of reported significantly higher levels of work–life stress, among whom the respondents who had childcare support (B = 0.409, *p* < 0.001) reported even more work–life stress than those who looked after their children by themselves or whose partners were the main caregivers (B = 0.294, *p* < 0.001). This was consistent with the findings for participants who had elderly relatives at home to take care of. Male participants who had elderly care support reported the highest level of work–life stress (B = 0.295, *p* < 0.001). Perceived availability of assistance was found to be negatively related to the level of work–life stress (B = −0.069, *p* < 0.01), and the effect was found to be higher among females (B = −0.112, *p* < 0.01). The overall model was significant, and the included predictors explained a significant amount of variance in work–life stress, R^2^ = 0.106, F = 11.36, *p* < 0.001.

In Model 3, participants’ satisfaction with their work life, family life, and the amount of time they spent at work and with their family were added into the regression analysis. The results showed that respondents’ satisfaction with their work life, family life, and the amount of time they spent at work and with their family were all negatively related to the level of work–life stress (Bs ranged from −0.108 to −0.429, all ps < 0.001). This finding applied to both the male and female respondents (Bs ranged from −0.087 to −0.438, all ps < 0.001), although the relationship between the male respondents’ satisfaction with family life and the level of work–life stress was not significant. The male respondents’ satisfaction with the amount of time they spent at work and with their family was most significantly related to the level of work–life stress (B = −0.483, *p* < 0.001). Results of the final model revealed that the work–life satisfaction measurement together accounted for a more significant proportion of variance in work–life stress, with R^2^ = 0.358, F = 40.79, *p* < 0.001.

## 4. Discussion

Our study examines the stress and satisfaction associated with work–life balance among dual-career individuals in Hong Kong and expands the literature with its special focus on the effects of individual-level, gender-specific determinants. Our study highlights that much attention should be paid to individual factors that relate to stress due to work–family imbalance, such as gender, long working hours, availability of family care assistance, and satisfaction with work–life demands. These demographic factors, influencing the perception in work–life balance, could serve as guidance in assisting policy formulation.

The respondents in our family survey encountered stress in balancing work and family life in general, and no gender difference was found in the scoring of work–life-balance-related variables, including the level of stress resulting from seeking to balance work and life, satisfaction with the amount of time spent at work and with family, and satisfaction with work life and family life. For females, the psychosocial burden of balancing long hours of work and housework is enormous [12,37]. Potential reasons for this prevalent problem include economic expansion, financial uncertainty, and inflexible working-time arrangements [10]. The power distance between employer and employee in Asian societies often makes employees less able to refuse to undertake additional hours, which imposes a serious work–life imbalance and has additional adverse impacts on well-being [38]. This could be explained by the notion that Chinese people possess a higher level of familism and collectivism that makes them view themselves as part of a larger social network, including work groups. In contrast, people holding individualistic beliefs would be unwilling to sacrifice their family time to work long hours and would put more emphasis on the need to separate work and life domains [15,33]. The Hong Kong Government has promoted family-friendly policies, such as a five-day workweek, paternity leave, and flexible working time, while less than half of all employees had access to these policies [25]. A recent evaluation report claimed that this could be explained by the voluntary nature of the policy implementation and employers’ uncertainty about the impacts of a family-friendly policy on work loyalty [25]. Our study suggested that employees from dual-career families still face high stress levels from work–life balance. Support from employers and the government is essential for implementing policies and organizational norms in reducing stress.

We found that participants with longer working hours reported higher stress levels from their work–life imbalance. The “Yerkes–Dodson law” suggested an inverted U-shaped relationship between quality of life due to stress arousal, where the peak accomplishment occurs by the stimulus of moderate-to-high levels of stress while the extremes of stress level could lead to a reduction in quality of life [39]. One of the causal pathways from long working hours to stress from work–life imbalance and morbidity is behavioral responses to stress through risky behaviors, such as tobacco and alcohol abuse and physical inactivity [5]. In this study, participants who were not in a marital or cohabiting relationship (i.e., divorced/separated, never married, or widowed) reported significantly higher levels of work–life stress. This finding is consistent with previous studies [37] that found that parents tend to frequently report the situation where long working hours in the office affects the parent–child relationship by causing the parents to often miss out on their children’s significant life events. The structure of the family has changed in recent years, including the diversified composition and scale of families, and this has led to the roles of family members becoming more complex [40]. For those whose are not married, work is often considered the central pillar of their identity, especially in the absence of a partner in the household [12]. Policymakers should focus more on these groups of individuals and families, by implementing practices, such as setting maximum working hours and extra paid leave, to gain greater control over work and life.

Gender differences were found in some parameters. Notably, we found that males’ satisfaction with the amount of time they spent at work and with their family was most significantly related to the level of work–life stress. This is similar to the findings in recent studies, which showed that satisfaction with work–life balance was slightly higher among men [3]. Our findings support the idea that women in contemporary families still take primary responsibility for managing the household and undertake parenting and childrearing responsibilities while working part or full time [3,41]. Previous research has indicated that long working hours for men might reinforce the male-breadwinner paradigm [42]. Some researchers [20] have also claimed that female managers report more stress and family problems than their male colleagues because they usually feel burdened by task-focused care within the family unit, which reversely contributes to work–life imbalance and low gender egalitarianism. We also found that males’ satisfaction with their family life and level of work–life stress was not significant, and males who had elderly care support reported the highest level of work–life stress. Some countries and districts have changed policies to facilitate a balance between work and family by offering more access to subsidized childcare or flexible working hours [43]. The welfare regimes in Nordic countries have been updating policies for compatibility between employment and personal life by promoting large investment in publicly provided childcare [3]. Further policy progress in Hong Kong could be achieved by conducting rigorous evaluation research to examine the effectiveness of and to set standards for effectively implementing family-friendly policies in the workplace by gender.

Our findings showed that the participants who had children and elderly relatives at home to take care of reported a significantly higher level of work–life stress compared to those who had no care duties, while the respondents who had care support reported even more work–life stress than those who looked after their children by themselves or whose partners were the main caregivers. This can be explained by the fact that in Confucian societies, family harmony is important, and so although having family support may contribute to family–work enrichment, a lack of support does not affect family–work imbalance [29]. The influence of Confucianism in Hong Kong and elsewhere in East Asia may also facilitate child-rearing support for the younger generation from family members, such as the able elderly [15]. Social support resources to help with housework or childcare often minimize detrimental outcomes by improving well-being and achieving work–life balance, which have been proven to be beneficial for reducing psychological strain and increasing satisfaction with work and life [19]. However, the rise of migrant domestic workers in developed areas, such as Hong Kong and Singapore, has been criticized by some researchers as an outcome of class-biased public policy that persistently overlooks the needs of lower-income families [44]. Therefore, the relationship between housework support and work–life balance among families of lower socioeconomic status under these marketization circumstances should be further examined.

Findings from this study should be interpreted with the following caveats. First, we employed data from a large family survey in Hong Kong for secondary analysis, and some of the explanatory variables in our study related to work–life balance were measured with a single item or self-constructed items and, therefore, may not assess the multiple perspectives of the work and life domains. Previous meta-analyses concluded that the single-item scale performed sufficiently well or sometimes even more robustly than multi-item measures on research topics, including job satisfaction [45] and quality of life [46]. Future studies could measure variables, such as the satisfaction and stress associated with work–life balance, by taking more perspectives into account. Second, we cannot establish a causal relationship between satisfaction with working hours and work–life balance due to the cross-sectional design of the study. We suggest that future research should design more follow-up studies to examine the longitudinal effects of demographic and family factors on work–life-balance-related characteristics. Third, we only included participants in Hong Kong to study the demographic and family characteristics related to work–life balance; no macro-level factors or cross-cultural comparisons were included in our study. Since work–life balance should be motivated by cultural beliefs and perceptions, it is suggested that future studies should focus on the effects of social and cultural predictors for evidence-based comparisons, in order to yield more rigorous results.

This study examined the associations between satisfaction with work–life balance and stress in balancing work and life through several demographic factors among dual-career families in Hong Kong. Previous studies revealed that most workers prefer more flexible working hours rather than material returns [47]. With a view to creating a more conducive environment for work–life balance, proactive steps should be taken to encourage employers to develop flexible employment practices and working conditions for employees. Moreover, the outbreak of the COVID-19 pandemic and lockdown measures are likely to make the boundary between working and home life less distinct. This has led some researchers to suggest that work–life balance should be examined under a different conceptual framework. Previous experience has shown that working hours increase dramatically after economic recessions, such as the Great Recession in 2008, which may also be the case after the COVID-19 pandemic [19]. Recent empirical studies also revealed a lower level of family functioning in families without children in the context of the COVID-19 pandemic [48]. As both exposed populations expand, the burdens of disease attributed to work–life imbalance may also increase [5]; this issue merits further investigation for timely prevention. The division of work among household members could also enter a new era of conflict after the pandemic due to work-from-home policies, where boundaries are extremely hazy. This poses huge challenges for future research related to work–life balance [12]. To expand our knowledge on how individuals in a particular place and time understand balance, more research is needed into how these meanings develop and how they impact individuals’ sense of entitlement to use “balance provisions,” such as flexible or reduced working hours, and their ability to access social support [49] to achieve balance, according to their perception of this concept.

## 5. Conclusions

The rapid economic growth in Hong Kong and many Asian districts and the increasing number of women in employment reflect the expansion of work opportunities, as well as the greater pressure on Hong Kong employees to balance work and family responsibilities. Individuals who manage to balance family and work life are more satisfied with their life, which positively impacts their mental and physical health. This will lead to a win-win situation, in which both employers and employees will benefit. On the other hand, work stressors have been developing rapidly, and yet there is a lack of formal policy support to facilitate work–life balance. In this study, using a representative family sample in Hong Kong, we discussed the network of individual and family variables that influence work–life constructs to guide future research. Our findings provide insight for future research to further investigate cultural and institutional contexts and their impact on work and life outcomes.

## Figures and Tables

**Table 1:** Demographic characteristics and explanatory variables of the participants.

N (%)	Total (n = 1300)	Men (n = 683)	Women (n = 617)	T-Test/ Chi–Square	*p*-Value
Age (Mean, SD)	44.37 (13.05)	44.76 (13.56)	43.93 (12.47)	1.156	0.248
15–34	702 (54.0)	376 (55.0)	328 (53.1)		
35–54	374 (28.8)	185 (27.2)	187 (30.3)		
55 or above	224 (17.2)	122 (17.9)	102 (16.5)		
Monthly income (individual, in Hong Kong Dollars)					
$9999 or below	239 (18.3)	69 (10.1)	169 (27.4)	85.353	<0.001
$10,000–$19,999	563 (43.3)	298 (43.6)	265 (42.9)		
$20,000–$29,999	228 (17.5)	154 (22.5)	74 (12.0)		
$30,000–$39,999	79 (6.1)	56 (8.2)	23 (3.7)		
$40,000–$49,999	24 (1.8)	15 (2.2)	9 (1.5)		
$50,000 or above	17 (1.3)	9 (1.3)	8 (1.3)		
No information provided	151 (11.6)	82 (12.0)	69 (11.2)		
Educational attainment					
Primary education or lower	167 (12.9)	80 (11.7)	87 (14.1)	2.150	0.341
Secondary education	786 (60.5)	424 (62.1)	362 (58.8)		
Postsecondary education or above	346 (26.6)	179 (26.2)	167 (27.1)		
Marital status					
Single (Divorced/separated, Never married, Widowed)	583 (44.9)	287 (42.1)	296 (48.1)	4.780	0.029
Married/cohabiting with a partner	714 (55.1)	395 (57.9)	319 (51.9)		
Have children	714 (54.9)	347 (50.8)	367 (59.5)	9.856	0.002
Have elderly relative aged 65+	798 (61.4)	413 (60.5)	385 (62.4)	0.509	0.475
Total number of working hours per week	44.90 (13.62)	47.59 (12.82)	41.91 (13.87)	7.618	<0.001
Major caregiver of children					
Child care support (Grandparent, relative, maids and others)	15.0% (n = 195)	12.7% (n = 87)	17.5% (n = 108)	11.619	0.009
Self/partner	24.3% (n = 316)	23.0% (n = 157)	25.8% (n = 159)		
Children do not need care provision	15.6% (n = 203)	15.1% (n = 103)	16.2% (n = 100)		
No children	45.1% (n = 586)	49.2% (n = 336)	40.5% (n = 250)		
Major caregiver of elderly aged 65+					
Elder care support (Relative, maids and others)	254 (19.5)	141 (20.6)	113 (18.3)	2.653	0.448
Self/partner	370 (28.5)	184 (26.9)	186 (30.1)		
Elderly do not need care provision	174 (13.4)	88 (12.9)	86 (13.9)		
No elderly relatives aged 65+	502 (38.6)	270 (39.5)	232 (37.6)		
Availability of assistance	4.40 (0.96)	4.35 (0.96)	4.45 (0.95)	−1.888	0.059
Level of stress resulting from efforts to meet the competing demands of work and family life (Mean, SD)	2.70 (0.74)	2.71 (0.74)	2.70 (0.75)	−0.174	0.862
Satisfaction with the amount of time spent at work and with family (Mean, SD)	3.60 (0.73)	3.59 (0.76)	3.62 (0.69)	−0.689	0.491
Satisfaction with work life (Mean, SD)	3.66 (0.63)	3.64 (0.63)	3.68 (0.63)	−1.070	0.285
Satisfaction with family life (Mean, SD)	3.89 (0.68)	3.92 (0.68)	3.86 (0.68)	1.780	0.075

**Table 2:** Associations of individual and family factors and stress from work–family balance.

	Total		Stress of Men		Stress of Women	
	Unstandardized Beta (95% CI)	*p*-Value	Unstandardized Beta (95% CI)	*p*-Value	Unstandardized Beta (95% CI)	*p*-Value
Model 1						
Demographics						
Age	0.000 (−0.004, 0.004)	0.994	0.001 (−0.005, 0.007)	0.632	−0.002 (−0.009, 0.005)	0.550
Monthly income	−0.015 (−0.064, 0.034)	0.554	−0.003 (−0.071, 0.064)	0.922	0.006 (−0.069, 0.082)	0.868
Educational attainment						
Primary education or lower	−0.034 (−0.222, 0.154)	0.720	−0.229 (−0.487, 0.030)	0.084	0.168 (−0.113, 0.449)	0.241
Secondary education	0.021 (−0.098, 0.140)	0.726	−0.006 (−0.165, 0.153)	0.938	0.077 (−0.106, 0.260)	0.410
Postsecondary education or above (reference)	1.00		1.00		1.00	
Marital status						
Single (Divorced/separated, Never married, Widowed)	−0.166 *** (−0.262, −0.069)	<0.001	−0.162 * (−0.306, −0.018)	0.028	−0.178 ** (−0.313, −0.044)	0.010
Married/cohabiting (reference)	1.00		1.00		1.00	
Total number of working hours per week	0.010 *** (0.006, 0.013)	<0.001	0.010 *** (0.005, 0.015)	<0.001	0.011 *** (0.006, 0.015)	<0.001
R^2^	0.043		0.053		0.050	
F	7.947		5.189		4.495	
df	6		6		6	
*p*-value	<0.001		<0.001		<0.001	
Model 2						
Major caregiver of children						
Child−care support (Grandparent, relative, maids, and others)	0.409 *** (0.274, 0.544)	<0.001	0.315 ** (0.111, 0.518)	0.003	0.471 *** (0.284, 0.658)	<0.001
Self/partner	0.294 *** (0.177, 0.411)	<0.001	0.297 *** (0.126, 0.467)	<0.001	0.280 ** (0.113, 0.447)	0.001
No children or no caring duties (reference)	1.00		1.00		1.00	
Major caregiver of children						
Child−care support (Grandparent, relative, maids, and others)	0.409 *** (0.274, 0.544)	<0.001	0.315 ** (0.111, 0.518)	0.003	0.471 *** (0.284, 0.658)	<0.001
Self/partner	0.294 *** (0.177, 0.411)	<0.001	0.297 *** (0.126, 0.467)	<0.001	0.280 ** (0.113, 0.447)	0.001
No children or no caring duties (reference)	1.00		1.00		1.00	
Major caregiver of elderly aged 65+						
Elder care support (Relative, maids and others)	0.227 *** (0.110, 0.344)	<0.001	0.295 *** (0.133, 0.457)	<0.001	0.141 (−0.028, 0.311)	0.102
Self/partner	0.127 * (0.023, 0.231)	0.017	0.111 (−0.038, 0.260)	0.145	0.147 (−0.001, 0.295)	0.052
No elderly aged 65+ or no caring duties (reference)	1.00		1.00		1.00	
Availability of assistance	−0.069 ** (−0.117, −0.022)	0.004	−0.031 (−0.100, 0.038)	0.383	−0.112 ** (−0.180, −0.045)	0.001
R^2^	0.106		0.103		0.133	
F	11.356		5.613		7.016	
df	11		11		11	
*p*-value	<0.001		<0.001		<0.001	
Model 3						
Satisfaction with the amount of time spent at work and with family	−0.429 *** (−0.488, −0.369)	<0.001	−0.438 *** (−0.517, −0.358)	<0.001	−0.417 *** (−0.508, −0.327)	<0.001
Satisfaction with work life	−0.180 *** (−0.247, −0.112)	<0.001	−0.197 *** (−0.292, −0.103)	<0.001	−0.168 *** (−0.266, −0.069)	<0.001
Satisfaction with family life	−0.108 *** (−0.170, −0.045)	<0.001	−0.087 (−0.176, 0.002)	0.055	−0.119 ** (−0.209, −0.029)	0.010
R^2^	0.358		0.381		0.351	
F	40.788		22.957		18.848	
df	14		14		14	
*p*-value	<0.001		<0.001		<0.001	

Note. CI, Confidence Interval; *p < 0.05. ** *p* < 0.01. *** *p* < 0.001. *p*-value by the likelihood ratio test. Variables in Model One were adjusted by other variables in the same phase, variables in Model Two were adjusted by all variables in Model One, and variables in Phase Three were adjusted by all variables in Model One and Model Two.

## Data Availability

Data available on request from the authors.
